# Epidemiological characteristics of patients who died of cancer in the Haydarpasa Training Hospital, Istanbul

**DOI:** 10.3325/cmj.2012.53.645

**Published:** 2012-12

**Authors:** Alpaslan Ozgun, Tolga Tuncel, Levent Emirzeoglu, Oguz Bilgi, Bulent Karagoz

**Affiliations:** Department of Medical Oncology, GATA Haydarpasa Training Hospital, Istanbul, Turkey

To the Editor: The October 2012 issue of the *Croatian Medical Journal* included an epidemiological study reporting cancer mortality in Turkey (1). Concerning this study, we would like to present epidemiological characteristics of patients who died of cancer in the period 2007-2010 at our center in Istanbul, as additional epidemiological data on cancer mortality in Turkey.

We retrospectively analyzed epidemiological characteristics of 255 cancer patients who died between July 2, 2007 and July 2, 2010 at GATA Haydarpasa Training Hospital, Department of Medical Oncology in Istanbul. The focus was on the distribution of the type of cancer depending on sex and age. In addition, a sub-group analysis was performed according to age groups. The most common causes of cancer deaths in men were lung cancer (41.5%), colorectal cancer (16.3%), and stomach cancer (7.5%) and in women breast cancer (27.1%), lung cancer (14.6%), and colorectal cancer (13.6%). When age groups were considered, the most common cause of death among men was Ewing's sarcoma between 20-40 years; lung cancer between 41-60 years and 61-80 years; and colorectal cancer in the group older than 81 years. The most common cause of death among women between 20-40 years, 41-60 years, and 61-80 years was breast cancer, while in the group older than 81 years it was lung cancer.

Lung and breast cancers are also most common causes of cancer death in the world (2-5). However, the third most common cause of death among men is prostate cancer, while in our study it was gastric cancer, with prostate cancer ranking fourth. The ranking of causes of death among women in our study was the same as in other studies in the world (2-5).

The number of studies on cancer epidemiology in our country is limited. In this regard, studies with larger number of patients that more accurately reflect the general characteristics of the patients in the country are necessary. In order to achieve this, it is necessary to establish a nation-wide and continually updated database.
